# The role of drug treatment and recovery services: an opportunity to address injection initiation assistance in Tijuana, Mexico

**DOI:** 10.1186/s13011-020-00322-1

**Published:** 2020-10-12

**Authors:** Stephanie A. Meyers, Claudia Rafful, Sonia Jain, Xiaoying Sun, Britt Skaathun, Andrew Guise, Patricia Gonzalez-Zuñiga, Steffanie A. Strathdee, Dan Werb, Maria Luisa Mittal

**Affiliations:** 1 School of Social Work, College of Health and Human Services, San Diego State University, 5500 Campanile Drive, San Diego, CA 92182 USA; 2 Division of Infectious Diseases and Global Public Health, Department of Medicine, University of California San Diego, 9500 Gilman Drive, MC 0507, La Jolla, CA 92093-0507 USA; 3 Facultad de Psicología, Universidad Nacional Autónoma de México, University City, Coyoacán, 04510 Mexico City, Mexico; 4 Centre for Urban Health Solutions, St. Michael’s Hospital, 30 Bond Street, Toronto, ON M5B 1W8 Canada; 5 Department of Family Medicine and Public Health, University of California San Diego, 9500 Gilman Drive, La Jolla, CA 92093 USA; 6 Addison House, Guy’s Hospital, King’s College London, Strand, London, WC2R 2LS UK; 7 Facultad de Medicina, Universidad Xochicalco, Rampa Yumalinda 4850, Colonia Chapultepec Alamar C.P, 22540 Tijuana, Baja California Mexico

**Keywords:** Medication assisted treatment, Methadone, Residential drug treatment, Injection drug use, Harm reduction, Tijuana, Mexico, Injection initiation

## Abstract

**Background:**

In the U.S. and Canada, people who inject drugs’ (PWID) enrollment in medication-assisted treatment (MAT) has been associated with a reduced likelihood that they will assist others in injection initiation events. We aimed to qualitatively explore PWID’s experiences with MAT and other drug treatment and related recovery services in Tijuana Mexico, a resource-limited setting disproportionately impacted by injection drug use.

**Methods:**

*PReventing Injecting by Modifying Existing Responses* (PRIMER) seeks to assess socio-structural factors associated with PWID provision of injection initiation assistance. This analysis drew on qualitative data from *Proyecto El Cuete* (ECIV), a Tijuana-based PRIMER-linked cohort study. In-depth qualitative interviews were conducted with a subset of study participants to further explore experiences with MAT and other drug treatment services. Qualitative thematic analyses examined experiences with these services, including MAT enrollment, and related experiences with injection initiation assistance provision.

**Results:**

At PRIMER baseline, 607(81.1%) out of 748 participants reported recent daily IDU, 41(5.5%) reported recent injection initiation assistance, 92(12.3%) reported any recent drug treatment or recovery service access, and 21(2.8%) reported recent MAT enrollment (i.e., methadone). Qualitative analysis (*n* = 21; female = 8) revealed that, overall, abstinence-based recovery services did not meet participants’ recovery goals, with substance use-related social connections in these contexts potentially shaping injection initiation assistance. Themes also highlighted individual-level (i.e., ambivalence and MAT-related stigma) and structural-level (i.e., cost and availability) barriers to MAT enrollment.

**Conclusion:**

Tijuana’s abstinence-based drug treatment and recovery services were viewed as unable to meet participants’ recovery-related goals, which could be limiting the potential benefits of these services. Drug treatment and recovery services, including MAT, need to be modified to improve accessibility and benefits, like preventing transitions into drug injecting, for PWID.

## Introduction

People who inject drugs (PWID) are disproportionately impacted by injection-related harms, like HIV, HCV, and overdose, and are at greatest risk for these outcomes within the first few years of initiating injection drug use (IDU) [1, 2]. This is likely due to novice PWID’s sharing of drug preparation equipment within social networks of more experienced PWID [1, 2], and the particular vulnerability of young PWID to punitive policy responses to IDU [3, 4]. Given these harms, it is imperative that efforts to mitigate the risks of injection-related harms among PWID are harmonized with efforts to prevent the initiation of vulnerable individuals into IDU [5], particularly in settings disproportionately impacted by injection-related HIV and overdose epidemics, such as Tijuana, Mexico.

### Injection drug use in Tijuana, Mexico

Estimates from 2018 suggest that there are approximately 12,000 PWID in Tijuana; an estimate that is over ten times the national average for Mexico [6, 7]. Tijuana is located along Mexico’s north western border, a region which has the highest level of risk for substance use in the country [8], and substantially more heroin use when compared to other regions of Mexico [9]. Tijuana has also been the site of a concentrated HIV epidemic driven by risk behaviors like IDU and sex work, as well as the scarcity of services addressing these behaviors. In addition, HIV prevalence in this area is estimated to be between 4.2 and 7.7% among PWID populations, and with approximately 30.5% of HIV cases among women [8, 10–13]. Furthermore, Tijuana serves as a key node along a drug trafficking corridor that supplies cocaine, opioids, and methamphetamine from Mexico into the United States and into Canada [14, 15]. It is therefore a critical site for exploring PWID’s experiences with drug treatment and recovery services, and how these experiences may influence drug injection trajectories, including the process of injection initiation.

### Medication-assisted treatment and the injection initiation process

Medication-assisted treatment (MAT) is the gold standard for treating opioid use disorders [16, 17]. MAT includes medications that are long-acting, full opioid agonists (i.e., methadone), partial agonists (i.e., buprenorphine), and antagonists (i.e., naltrexone), and serve to relieve the withdrawal symptoms and psychological cravings that accompany opioid use disorder [16]. Medication-assisted treatment (MAT) enrollment has also been shown to reduce opioid use frequency and street-based injecting among PWID [18, 19], and recently, MAT enrollment has also been shown to be associated with a lower likelihood of PWID providing injection initiation assistance in both San Diego, USA and Vancouver, Canada [20, 21]. It is hypothesized that MAT has this secondary preventative impact on providing injection initiation assistance through the disruption of drug use processes, like non-injection opioid use and street-based injecting, that place PWID at risk of being asked to provide injection initiation assistance [4, 21]. These findings highlight MAT’s potential role as a tool for both the treatment and prevention of opioid IDU.

### Drug treatment and recovery services in Tijuana

Despite the aforementioned MAT-related benefits, however, the cost of MAT provision only (i.e., excluding other social services) has previously been cited by PWID as a significant barrier to accessing these services [22]. There have been up to four operating MAT clinics in Tijuana [23]. During the time of the study, there were two operating MAT clinics, one private (450 patients/year) and one public (216 patients/year), with the latter receiving 95% of their operating budget through government subsidies [24]. PWID living near the San Ysidro US-Mexico border crossing would require a 10–15 min walk to the private clinic and a 2 h walk up a 167 m hill to the public clinic [25]. The cost of MAT ranges from USD$2.81 to $5.59 per client in Tijuana, whereas the cost of an average dose of heroin is roughly $1.30 in this setting (though PWID often need multiple doses of heroin in a day), indicating MAT costs could be prohibitive and serve as a substantial barrier [24].

There is wide variability among available drug treatment and recovery service options in Tijuana, outside of MAT-related services, similar to what has been found in other regions of Mexico, as well as in Latin American and Asian countries [26–28]. For example, clients in Tijuana may be in drug treatment on a voluntary or involuntary basis [26], with involuntary treatment being defined as the mandatory enrollment of individuals, who may or may not be drug dependent, in a drug treatment program [26, 29]. Involuntary treatment, however, has primarily been found to be limited in helping individuals achieve their recovery-related goals (e.g., reducing frequency of use, avoiding overdose, avoiding withdrawal, and avoiding injection-related risks [including injection initiation events]) and some research that has shown this modality to be both a human rights violation and associated with an increased risk of non-fatal drug overdose for PWUD in Tijuana [29, 30].

Furthermore, the majority of people who use drugs (PWUD; including PWID) that receive drug treatment and recovery services in Tijuana, do so at centers, referred to as *anexos*, that are privately owned and operate outside of government oversight [26, 31]. Less than half of drug treatment centers in Mexico are certified by the Comisión Nacional contra las Adicciones (CONADIC; Mexican National Commission against Addiction), and therefore may be offering services lacking the minimal criteria for quality care [23]. The lack of governmental oversight for drug treatment and recovery service centers in this region has been associated with overcrowded conditions, the absence of medical services, abstinence-based withdrawal practices, and fear of violence among clients [26, 31, 32]. Most of these centers employ a “mutual aid” approach, are faith-based, and are based in the Alcoholics Anonymous (AA) 12-step model of drug treatment [26, 33]. In AA and narcotics anonymous (NA) treatment models, in Tijuana and other North American regions, MATs, specifically, are not approved treatments and are, therefore, not applied to help clients manage withdrawal [34]. The medications offered in these *anexos* could include, however, benzodiazepines like clonazepam and dextropropoxyphene, to help clients manage their anxiety [35]. Additionally, it has also been estimated that retention within these faith-based treatment centers is approximately 39% in Tijuana, which indicates its inability to retain PWUD and aid PWUD in achieving their recovery-related goals in this setting [33].

There is documented variability in available drug treatment and recovery services, including MAT-related services, in Tijuana. As such, research is needed to fully explore experiences with, and the influence of, differing treatment modalities (i.e., involuntary treatment, voluntary treatment, and methadone treatment) on PWID’s ability to achieve their recovery-related goals among PWID in Tijuana. Consequently, the current qualitative study seeks to expand the existing literature by addressing the following aims: (1) to explore the experiences of PWID in accessing differing drug treatment and recovery services, including MAT, and (2) to explore how experiences with drug treatment and recovery services may influence their ability to achieve recovery-related goals (i.e., reduce their frequency of use, avoid withdrawal, avoid overdose, and reduce injection-related risks [including injection initiation events]) in the resource-limited context of Tijuana, Mexico.

## Materials and methods

### Study characteristics

Data for the present study were drawn from *PReventing Injecting by Modifying Existing Responses* (PRIMER; NIDA DP2-DA040256–01), a multi-cohort study seeking to investigate whether interventions to reduce injection-related HIV risk may be effective in preventing the provision of injection initiation assistance by PWID, in recognition of the role of PWID in facilitating the vast majority of injection initiation events [4, 5]. The methods for PRIMER in Mexico, U.S., Canada, and France, have previously been described in full [5]. The present PRIMER investigation includes qualitative data collected in September 2016 from the Tijuana-based *Proyecto El Cuete IV* (ECIV) subcohort. For the present study, PRIMER participants were enrolled in the ECIV study, ≥18 years old, reported IDU in the 30 days prior to ECIV baseline, were a resident of Tijuana and planned to remain in the area for at least 24 months, and were fluent in either English or Spanish. Based on ECIV participants’ quantitative reports of injection initiation assistance provision, MAT enrollment, and a history of incarceration, a purposive sample of ECIV participants that met the aforementioned PRIMER inclusion criteria were recruited for semi-structured qualitative interviews [36]. Additionally, to examine the social norms and stigma associated with helping others to inject, a sub-sample of those participants who did not report assisting others was also included in the qualitative interviews [36]. All qualitative interviews were conducted in English or Spanish between June and September of 2016 in Tijuana [36]. PRIMER and ECIV both received approval from the University of California, San Diego Institutional Review Board (IRB) and the Universidad Xochicalco Ethics Committee.

### Qualitative data collection

In-depth qualitative interviews were conducted with a subset of ECIV study participants and included open-ended questions and prompts to explore participants’ current life situation, experiences of providing injection initiation assistance, and potential preventive interventions for injection initiation, including MAT and other drug treatment and recovery services [36, 37]. All interviews were conducted in English or Spanish by social science researchers (AG, CR, and MLM) with previous qualitative research experience with communities of PWUD. These interviews took place in offices in Tijuana that were familiar to participants and sought to explore the topics of interest while allowing participants to elaborate in their own words. Interviewers did not probe further on interview topics if contradictions emerged in their accounts or if verbal or non-verbal cues indicated an unwillingness to discuss the subjects raised. The interviews ranged from 20 to 90 min in length but lasted an hour, on average. Interviews were transcribed and translated to English when needed. Additionally, all participants recruited for the qualitative interviews received USD$25 compensation for their time and travel costs. All names presented herein are pseudonyms to preserve participant confidentiality.

### Analyses

In-depth qualitative interviews were coded and analyzed thematically by a team of six social scientists. For the current analysis, codes were developed both in response to the quantitative findings, and by drawing on existing concepts from the literature, to assess experiences of abstinence-based drug recovery services, MAT enrollment, and providing injection initiation assistance [38]. Additionally, we developed themes from meaningful data and iteratively refined them through multiple coding stages [38, 39].

## Results

A total of 748 PWID from ECIV completed a quantitative PRIMER baseline between 2014 and 2017 (Table 1). Of the PWID sampled, 81.1% reported daily IDU in the past 6 months and 5.5% reported providing injection initiation assistance in the past 6 months. In terms of recent (past 6 month) drug treatment and recovery service enrollment, 6.4% reported enrollment in voluntary treatment, 3.1% reported enrollment in involuntary treatment, and only 2.8% reported enrollment in MAT (i.e., methadone only; Table 2), indicating relatively low levels of recent drug treatment and recovery service uptake for PWID in this setting. See Tables 1 and 2 for a summary of the descriptive statistics.

**Table 1:** Descriptive characteristics of PRIMER participants that completed the quantitative baseline survey in Tijuana, Mexico (*n* = 748)

***Categorical Variables***	***n (%)***
*Gender*	
Men	457 (61.1)
Women	291 (38.9)
*Housing Status*	
Stable Housing	465 (62.2)
Other	283 (27.8)
*Marital Status*	
Married	326 (43.7)
Other	420 (56.3)
*Injection Drug Use Frequency*^a^	
Daily	607 (81.1)
Less Than Daily	41 (5.5)
None	100 (13.4)
*Provided Injection Initiation Assistance*^a^	
Yes	41 (5.5)
No	707 (94.5)
*Enrolled in any Drug Treatment or Recovery Service*^a^	
Yes	92 (12.3)
No	656 (87.7)
*Voluntarily Enrolled in Abstinence-Based Drug Recovery Services*^a^	
Yes	48 (6.4)
No	700 (93.6)
*Enrolled in MAT*^a^	
Yes	21 (2.8)
No	727 (97.2)

^a^The variable refers to activities during the previous six months; MAT: Medication-Assisted Treatment; SD: Standard Deviation

**Table 2:** Descriptive statistics for drug treatment and recovery services and the provision of injection initiation assistance among study PWID who completed the PRIMER baseline survey in Tijuana, Mexico (*n* = 748)

	Past 6 Month Injection Initiation Assistance	
Events	No Frequency (Column %)	Yes Frequency (Column %)
*Any Drug Treatment or Recovery Service*^a^		
No	621 (87.8)	35 (85.4)
Yes	86 (12.2)	6 (14.6)
Total	707 (100)	41 (100)
*Drug Treatment or Recovery Service*^a^		
None	621 (87.8)	35 (85.4)
MAT	20 (2.8)	1 (2.4)
Voluntary Abstinence-Based Recovery Services	45 (6.4)	3 (7.3)
Involuntary Abstinence-Based Recovery Services	21 (3.0)	2 (4.9)
Total	707 (100)	41 (100)
*MAT Enrollment*^a^		
No	687 (97.2)	40 (97.6)
Yes	20 (2.8)	1 (2.4)
Total	707 (100)	41 (100)

^a^The variable refers to activities during the previous six months; MAT: Medication Assisted Treatment

Of the 21 ECIV participants that underwent qualitative interviewing, 13 (61.9%) self-identified as male. The mean age of the participants was 40.6 years (range: 25–60). Eleven (52.4%) participants reported experiences with drug treatment and recovery services in their lifetime and 14 (66.7%) of the participants reported MAT enrollment experiences in their lifetime. As such, this qualitative analysis focuses on these participants.

Two themes emerged within the qualitative data regarding experiences with abstinence-based drug recovery services: (1) its limitations in meeting clients’ recovery-related goals and (2) negative experiences and abuse within this treatment modality. Furthermore, two themes specifically related to MAT enrollment emerged: (1) individual barriers and (2) structural barriers.

### The limits of abstinence-based drug recovery services

Nine participants (42.9%) described their experiences using abstinence-based drug recovery services as cyclical in nature. In this abstinence-based drug recovery service cycle, participants recounted either voluntarily attending, or involuntarily being committed to, a variety of rehabilitation centers for durations of just one to two months before departing. Mirroring past research [26], participants perceived treatment limitations given their difficulty in completing the recovery service programs and attaining long-term sobriety goals extending past their participation in the services. As a result, participants described experiencing burnout after rotating in and out of recovery services multiple times. This dynamic is exemplified by Omar, who recalls his experience with voluntary rehabilitation:

“*It was hard cause um, ain’t the first time I go to rehab, it was about 6 times that I tried to uh … quit uh heroin and the other drugs that I’m using. So, it was hard cause um*, la malilla [dopesickness]*. We call it* la malilla *when you feel sick when you aren’t using these drugs in the rehab. You start to feel sick and and and sometimes they give you pills* [sedatives] *and …*. *No, they didn’t give me pills and I was craving it and, and then I tell the, the guards over there and the director over there, uh, that I want to leave, they said ‘okay you are free to leave because we don’t, we cannot have you here forced’.*” (Omar, 47).

Omar’s account highlights the variability across abstinence-based drug recovery services in how they seek to attenuate withdrawal in the absence of opioid agonist or antagonist treatment options (e.g., methadone). This variability and lack of withdrawal management impacts participant retention in abstinence-based drug recovery services and calls into question their ability to meet client’s recovery-related goals, particularly in cases where MAT (i.e., the gold standard for the treatment of opioid use disorder) is not provided [18, 19].

In addition, participants also reported witnessing substance use within abstinence-based drug recovery service centers and expressed beliefs that the mismanagement of these centers was to blame. The following narrative from Leticia depicts an experience with an abstinence-based drug recovery service center in which other clients brought and used substances on the premises:

*“But I mean there’s places here that they put on the street for rehabs, it’s a bunch of bullshit. People come from other sites to rehab right here to Tijuana, they bring their ounce of coke, a gram of heroin, so when they are kicking, and every day they get high, even the supervisor is providing the pills for them, just when visits come around they clean up the place, and they make it good, you know? So the family could give more money to the place.”* (Leticia, 41).

This account indicates that there may be abstinence-based drug recovery services that lack structured treatment plans, which consequently allow some clients to bring substances into the center and creates unclear expectations regarding the services offered. As a result, it appears that the substance use on behalf of those seeking services serves to negatively color Leticia’s perception of the medications (i.e., pills) administered by staff at the center, and even the validity of the rehabilitation center itself to meet her recovery goals. This depiction further contributes to the broader pattern of the of abstinence-based drug recovery services’ inability to meet clients recovery-related goals.

Furthermore, limited accounts (i.e., two participants) described injection initiation events occurring within social connections that were shaped by, or formed in, these centers. For example, these accounts from Bryan and Martina illustrate how social connections, abstinence-based drug recovery service experiences, and injection initiation can be intertwined in this setting:

*“Mm (chuckles). I met her at a rehabilitation center.* [Name of rehabilitation center]*, um, well we were there, and in fact I went for her when, when, when her mom put her there. She put her in* [name of rehabilitation center] *and I went for her, I got annoyed with her because she behaved very... and rude. And well the only thing we wanted was* [for me] *to take her and do her the favor* [provide injection initiation assistance] *so that she could feel better.”* (Bryan, 35).

*“Well he, to see if he could understand me, that because he would see me and he would say that he would see me a lot and he wanted to see why I couldn’t stop that [substance use], because he sent me to centers many times and um … well no, I could not do … I would last a month and I would go back to the same and he wanted to know why, why I would go back to that, he wanted to understand me and that the best way to understand me was for him to use* [to initiate IDU]*. To see if I … to see if it would help me and I would leave everything …*” (Martina, 38).

Bryan met a woman in an abstinence-based drug rehabilitation center who had previously smoked crystal methamphetamine and was in treatment involuntarily. This woman subsequently became Bryan’s intimate partner, and when she was able to leave, Bryan provided her with substances and injection initiation assistance in an effort to manage her drug craving. Martina had experienced a cyclical relationship with abstinence-based drug recovery services that had failed to help her meet her recovery-related goals. This inability to help Martina manage her substance dependence ultimately caused tension between Martina and her partner, and her partner ultimately requested assistance with initiating IDU to increase their level of intimacy. This corresponds to past qualitative research that has illustrated PWID provide injection initiation assistance for a variety of reasons, including the desire to share the drug use experience and the high, to increase intimacy and/or relationship satisfaction, to counteract a partner’s increasing tolerance and/or the economic cost of drug use, and in an effort to protect novice initiates in the face of an opioid overdose epidemic [37, 40, 41]. Additionally, though gender did not emerge as a theme in these accounts, these narratives correspond to past literature highlighting that injection initiation often occurs within the social context of intimate partnerships, and that gendered power dynamics can exist within injection initiation processes, particularly for women in Tijuana [42, 43]. The current study, however, expands these findings to highlight that the lack of evidence-based drug recovery services for Bryan and Martina failed to treat their substance dependence and meet their recovery-related goals, as evidenced by these services’ influence on their and their intimate partner’s injection initiation processes. These experiences, therefore, further illuminate the intertwining of abstinence-based drug recovery services and social connections, and how this intersection could potentially increase the risk of injection initiation events.

### Negative experiences and abuse within abstinence-based drug recovery

In addition to the experiences of abstinence-based drug recovery service variability and reported inability to meet recovery goals, five participants (23.8%) also reported either negative perceptions of, or adverse experiences with, abstinence-based drug recovery service centers. This is illustrated through Luna’s experiences with involuntary treatment within these recovery service centers:

“*… Here* [Tijuana] *you don’t have rights, on the other side* [the United States] *you do, but not here … over there it is* [considered] *kidnapping … not here. Right here you can’t say anything because you are an addict, you no longer think good, you no longer have a good opinion, like they say you don’t … you don’t have rights anymore. It seems like a little absurd to me but … at my age, my family, every time they can they try to send me to a rehabilitation center and I can’t do anything. And they can leave for as long as they want and I can’t do anything. Even if I am over age. They go for you to your house, they pull you out, they take you to a rehabilitation center and once inside you can no longer do anything.*” (Luna, 25).

This suggests that client mistreatment and the denial of human rights may be common experiences within involuntary abstinence-based drug recovery centers for PWID in Tijuana. This account further contributes to the broader pattern of participants’ negative experiences with abstinence-based drug recovery services, and their inability to meet clients’ recovery-related goals.

#### Individual barriers to MAT use

In regards to PWID’s experiences with evidence-based drug treatment (i.e., MAT services), several individual level barriers were identified in the participant narratives that may be limiting the ability of MAT programs to meet participant needs in Tijuana. Indeed, five participants (23.8%) expressed ambivalence regarding MAT services in Tijuana, where these primarily take the form of outpatient methadone clinics. There are three of these methadone clinics (two private and one public) available in Tijuana, and the number of PWID in this region far outnumber the capacity of these providers [24]. Individual ambivalence is illustrated in the following narrative in which Israel simultaneously describes methadone as both positive and negative:

“*I mean it helps out it helps out a lot you know. It helps you function in society you know* (laughs) *unlike heroin you know when you wake up in the morning and you’re like,* “ohh man” *and your whole body aches you know if you don’t have it you ain’t going to work, you ain’t getting out of bed, you ain’t gonna do nothing till you get that fix. Unlike with methadone, you know when you take it and you wake up in the morning you still have that effect, you know, where you don’t feel that sickness or nothing so you are able to wake up normal you could say you are able to function. I don’t, I don’t recommend it for anybody ‘cause it kills you faster than heroin and the sickness is a lot worse than just heroin itself. Cause I’ve kicked methadone before and it’s nothing nice … It’s nothing nice.*” (Israel, 44).

In the above account, Israel described methadone as both something that allows people to manage opioid use disorder while also simultaneously being worse than heroin. This ambivalence is seemingly driven not only by the capacity of methadone to curb drug craving and to facilitate daily functioning, but also by narratives of side effects that accompany its use. Similar to studies in Iran, China, and Norway [44–46], five (23.8%) participants reported difficulty with swelling, liver dysfunction, and weight gain known to the community to be associated with methadone. This implies a need for increased dissemination of knowledge on MAT and possible side effects. Additionally, ambivalence towards MAT could contribute to the discontinuation or inconsistent use of MAT, thereby reducing the beneficial impact of this form of treatment.

Seven participants (33%) also reported holding negative stereotypes of methadone use. As in Israel’s account, a commonly held negative stereotype of methadone is that it is either the same as, or worse than, other substances in terms of dependence. Lucas’ description of methadone typifies this stereotype:

“*I have seen people, that is more addicted to methadone, and suffer more than with heroin. That is what I know, they get really cruel withdrawals, their whole-body hurts, and that well, the government tolerates it because they know that they are getting money.*” (Lucas, 50).

These stereotypes and negative experiences expressed by Lucas and other participants in this sample, echo what has also been found in qualitative research with PWID in the United States. The belief that methadone is a “nasty” and “cruel” treatment that is as bad as other substances is persistent [22, 47, 48]. These negative perceptions of methadone use could be, in part, due to poor management of opioid use disorders in MAT clinics, as well as experiences with interrupted service access and with methadone withdrawal. Employing patient-centered approaches to MAT enrollment, like low or no cost and mobile MAT services, have been found to help maintain MAT-related retention, which could potentially buffer against negative perceptions of this treatment [49]. These patient-centered approaches, however, currently do not exist in Tijuana [24]. As a result, the negative perceptions PWID expressed can further act as a barrier to accessing MAT, which could be limiting the ability of MAT enrollment to help PWID in Tijuana meet their recovery-related goals.

The concurrent use of other substances (i.e., opioids or methamphetamine) while using methadone also appeared to be a barrier to PWID achieving recovery-related goals. Eleven participants (52.4%) reported either using substances in conjunction with methadone or hearing of ubiquitous substance use among those in methadone treatment. Some participants also reported that encountering other people selling substances while waiting in line for methadone was a common occurrence. Here Lucas recalls his experience with concurrent substance use while on methadone:

“*So I came over here to the center and, and I learned about that* [methadone]*. And I went, I tried it, but they told me... Take the juice, and after about 20 minutes when I got to the center, I got the urge to inject, and I injected, and well I kept going. So I said,* “well there is no need to, to keep taking the juice and still drug myself.” *So, 2, 3 days passed, and I went again and I said,* “no, I’m not going to go because I am wasting it [methadone] anyways, so I better not.” *Because over there* [at the methadone center] *they told me,* “no, you cannot do that, use methadone and inject because it can really harm you.” *Well nothing happened to me, so I said,* “no, it’s better if I just do one thing.” *And besides over there* [at the methadone center] *they sell you, they sell you pills.*” (Lucas, 50).

Lucas recounts still experiencing drug cravings while on methadone and ultimately feeling like it may be unsafe or wasteful to continue the concurrent use of other substances with methadone. Additionally, similar to Leticia’s account of substance use in abstinence-based drug recovery service centers, Lucas seemed to view the use of prescribed psychotropic medications provided at the methadone center as akin to non-prescription substance use by stating, “they sell you pills.” It is possible that non-patient-centered approaches and interrupted MAT service access or inadequate MAT dosages could be facilitating PWID’s concurrent use of substances with methadone. Furthermore, as Lucas depicted, PWID’s concomitant substance use could serve as an additional barrier to accessing MAT and achieving recovery-related goals.

Lastly, MAT enrollment did not seem to alter participants’ social drug-using connections. The following narrative from Luna depicts how she still interacted within a substance use-based social group while enrolled in MAT, which could potentially interfere with her recovery-related goals by increasing the risk of injection initiation events [50]:

“*Not the last time, but out of the times I was on methadone I ran into a friend, an acquaintance who was an ex-boyfriend of a friend. I had just medicated* [taken methadone] *and the guy was … I don’t know what he had or why I ran into him on methadone, the point is that he asked me for the favor to connect and … And well I don’t know, he had already injected before, I just helped his girlfriend who had already injected before but did not know how to inject herself, and he wanted to get fixed and he told me,* ‘I’ll fix you.’” (Luna, 25).

Luna’s experience shows how drug using relationships can persist even when participants are accessing methadone services. Furthermore, continued social interactions with substance using contexts can act as a barrier to the potential benefits of MAT enrollment and potentially negatively impact PWID’s ability to achieve their harm reduction goals by increasing their risk for injection initiation events.

#### Structural barriers to MAT enrollment

Four participants (19%) identified structural-level barriers to MAT enrollment, including issues with the cost, crowding, time commitment, and geographic locations for MAT. The following narrative from Omar illuminates these barriers:

“*We used to have three methadone clinics over here, but now we only have two, but one is not taking people which is too crowded right now. And the other one is methadone they made it over there* [far away] *and it’s not very, nobody likes to go over there so … It’s not very good. They they they ha they should’ve had … more original* [pre-markup] *prices because they are very high. In the price. Somebody who makes two hundred pesos a day* [<$10 USD] *has to pay uh um eighty-seven pesos* [<$5 USD] *for a methadone a day, so … They they better pay a hundred pesos* [~$5 USD] *for heroin and and and and cut it on that day.*” (Omar, 47).

Omar’s account is in-line with past research that has found that methadone treatment in resource-limited settings can often be expensive, time intensive, and physically hard to access [44, 46]. These structural barriers to methadone and other MAT services use, then, could be further minimizing the beneficial effects of this form of treatment and reducing PWID’s ability to attain their recovery-related goals within this setting.

## Discussion

We found low levels of MAT and other drug treatment and recovery service enrollment among PWID in Tijuana. The narratives reported herein, however, help illuminate participants’ experiences with drug treatment and recovery services within this setting, and demonstrate that there may be a lack of consistent, evidence-based drug treatment options for PWID in Tijuana. These findings build on past research, which has found that drug treatment and recovery services in Tijuana are often overcrowded, lack medical services, lack state oversight, and provide limited to no access to MAT or psychosocial treatment [23, 26].

Furthermore, involuntary drug treatment services have largely been found to be unable to meet client’s recovery-related goals in this setting and others, and in some cases are even harmful, for PWID populations [26, 29]. Narratives of negative experiences from participants corroborate literature demonstrating that mistreatment in the form of physical and verbal abuse can be common experiences within involuntary, abstinence-based drug recovery centers for PWID in Tijuana [32, 35] and other parts in Mexico [28]. Furthermore, the variable quality and reported dissatisfaction with abstinence-based drug recovery services, as well as the lack of access to evidence-based treatment options in Tijuana, could be minimizing any potential effect these drug treatment and recovery services may have on helping participants’ achieve their recovery-related goals, including reducing the risk of injection initiation events.

Additionally, experiences with abstinence-based drug recovery centers and methadone enrollment in Tijuana did not serve to change PWID’s substance-using social connections. Many participants recounted either continued socialization within existing substance use-based social connections, or being introduced to new substance-using individuals, within the contexts of abstinence-based drug recovery services and MAT enrollment. In limited instances, participants were presented with requests to provide injection initiation assistance by the individuals they met in abstinence-based drug recovery services, which highlights that these services may also influence injection drug use initiation events, both by injection-naïve individuals and PWID who may assist in this process.

Lastly the abstinence-based recovery and withdrawal practices within many of the rehabilitation centers in Tijuana may be further diminishing the potentially beneficial impact of MAT for PWID. For example, preliminary research from Vancouver found that a higher proportion of those PWID that perceived their MAT dose to be “too low” also reported providing injection initiation assistance when compared to those that perceived their MAT dose to be “adequate” or “high” [21]. Participant narratives demonstrating continued drug cravings and concurrent substance use while enrolled in MAT may indicate that there is interrupted, inadequate, and inconsistent MAT availability for PWID in treatment in Tijuana, which could further limit the beneficial effects of this treatment on participants’ ability to attain recovery-related goals and on reducing the risk of injection initiation events.

### Limitations

Study limitations may include limited representation of the broader PWID population in Tijuana. We also note that the target population of interest is mobile and difficult to access in Tijuana where PWID face vulnerabilities related to violence, barriers to accessing health care services, and punitive policing practices [12, 51, 52]. Furthermore, given that participants’ current substance use was part of the inclusion criteria for the ECIV study from which participants were sampled in 2011, participant selection for PRIMER qualitative interviews may be biased against those with positive drug treatment and recovery service experiences. However, this ongoing cohort study does not exclude participants that are not currently using substances. Moreover, the qualitative interview guides, though they were designed to elicit responses regarding experiences with abstinence-based drug recovery service and MAT enrollment, also explored a range of experiences related to the provision of injection drug use initiation. For many participants, the concept of injection initiation assistance was new, and although we made every attempt to develop their knowledge, our efforts may have been insufficient. Some participants grasped the concept quickly, labeling the person they assisted as a *primerizo* (first-timer) [36], and others required additional probing to discern how individual experiences of drug treatment influenced their decision to provide injection initiation assistance to injection-naïve individuals versus persons who had injected before they met at the drug treatment center. Consequently, the findings presented are exploratory in nature and should be interpreted cautiously. Lastly, the subject of IDU initiation is sensitive in nature, and the reliance on self-report within this study could lead to response bias and the underreporting of initiation behaviors [37, 53].

## Conclusion

The findings from the current study are critical for informing drug treatment and recovery service efforts that may, alongside helping PWID in Tijuana achieve their recovery-related goals, also reduce their risk of injection initiation events. The results from this study, however, indicate that available abstinence-based drug recovery service centers and MAT clinics will need to be redesigned in order to make these treatment modalities accessible and beneficial for PWID. In order to better serve the needs of PWID in this region, it is also recommended that patient-centered approaches to MAT be employed. Patient-centered approaches have demonstrated effectiveness in increasing MAT-related retention, and could potentially help to alleviate some of the structural barriers related to overcrowding and patient cost within this setting [49]. It is also recommended that behavioral interventions such as Break the Cycle or Change the Cycle be employed to reduce the likelihood of PWID engaging in injection initiation processes [4, 54]. Indeed, MAT and other drug treatment and recovery services could be potential sites for the implementation of these interventions. Lastly, we advocate for MAT to be operationalized as a harm reduction approach for reducing the risk of injection initiation events and other injection-related harms.

## Data Availability

The datasets generated and analyzed during the current study are not publicly available due to [reason] but are available from the corresponding author on reasonable request.

## Abbreviations

AA: Alcoholics anonymous
ECIV: Proyecto el Cuete IV
IDU: Injection drug use
HIV: Human immunodeficiency virus
MAT: Medication assisted treatment
NA: Narcotics anonymous
PRIMER: PReventing injecting by modifying existing responses
PWID: People who inject drugs
PWUD: People who use drugs
